# Prevalence and characteristics of thelarche variant

**DOI:** 10.3389/fendo.2023.1303989

**Published:** 2023-12-01

**Authors:** Francesca Burlo, Beatrice Lorenzon, Gianluca Tamaro, Antonella Fabretto, Francesca Buonomo, Martina Peinkhofer, Viviana Vidonis, Giada Vittori, Elena Faleschini, Egidio Barbi, Gianluca Tornese

**Affiliations:** ^1^ Department of Medicine, Surgery and Health Sciences, University of Trieste, Trieste, Italy; ^2^ Institute for Maternal and Child Health IRCCS “Burlo Garofolo”, Trieste, Italy

**Keywords:** endocrinologic diseases, stimulation tests, epidemiology, precocious puberty, thelarche premature, FSH & LH, thelarche variant

## Abstract

**Introduction:**

Girls with early thelarche may show an intermediate clinical picture between isolated premature thelarche (PT) and central precocious puberty (CPP), defined as “thelarche variant” (TV), characterized by an FSH-predominant response, although a univocal definition is lacking.

**Methods:**

Retrospective analysis on 91 girls with early thelarche (<8 years) and advanced bone age and/or accelerated growth who underwent 104 LHRH tests. Patients were classified into CPP (LH peak ≥5 IU/L; n = 28, 31%), TV (FSH peak ≥20 IU/L, LH peak <5 IU/L; n = 15, 16%), or PT (FSH peak <20 IU/L and LH peak <5 IU/L; n = 48, 53%).

**Results:**

TV patients were younger (5.51 years) and with less advanced bone age (+0.8 years). They had higher basal and peak FSH (2.5 and 26.6 IU/L) and lower basal and peak LH/FSH ratios (0.08 and 0.11). The prevalence of presence of ovarian follicles >5 mm in TV (42%) was similar to CPP but significantly higher than PT, whereas maximum ovarian volume was smaller in TV (1.0 cm^3^). At the last follow-up visit (available in 60% of the cases), 44% of TV developed CPP compared with 14% of PT (p = 0.04). At first evaluation, those who progressed to CPP had a higher basal FSH (3.2 IU/L), lower LH/FSH ratio (0.07), and a higher peak LH (4.1 IU/L) compared with those who did not progress to CPP (basal FSH 1.9 IU/L, p < 0.01; basal LH/FSH ratio 0.12, p < 0.01; peak LH 2.8 IU/L, p = 0.02).

**Conclusion:**

Using laboratory parameters only as a definition, we identified the clinical, laboratory, and imaging features of TV: these girls showed less advanced bone age and FSH predominance also at baseline, with smaller ovaries but with follicles >5 mm. Almost half of girls initially diagnosed as TV developed CPP at last follow-up visit, and these girls had higher baseline FSH, lower baseline LH/FSH ratio, and higher peak LH at first evaluation. Therefore, TV may represent a “precocious prepuberty” in which the FSH predominance may initially limit the progression into proper puberty, but it may eventually trigger full puberty (even CPP, depending on the girls’ age).

## Introduction

Early sexual maturation in girls is defined as the development of breast buds under the areola, also known as thelarche (Tanner stage 2), before the age of 8 years (1–3), and it is one of the most frequent causes of pediatric endocrine referral (4). Several conditions could determine early breast budding, including central precocious puberty (CPP) (gonadotropin-dependent) and precocious pseudopuberty (gonadotropin-independent), in which thelarche is associated with growth acceleration, advanced bone age, and increase in uterine size due to estrogen exposure (5), but also isolated premature thelarche (PT) (6). This is a common and usually self-limiting condition without any other sign of sexual maturation that does not influence the growth or the timing and progression of puberty (7); clinical monitoring of pubertal progression and growth is recommended, as it helps differentiate this condition from CPP in most cases (8).

However, in clinical practice, some girls with an early thelarche may show an intermediate clinical picture between isolated PT and CPP. This condition has been defined over the years as “thelarche variant” (TV), but also “slowly progressive variant of precocious puberty in girls,” or “exaggerated thelarche”; the terms have been used interchangeably as synonyms, and several clinical cases have been reported (9–13) (**Table 1**).

**Table 1:** Summary of thelarche variant definitions in the literature.

Definition	Features	Ref.
Thelarche variant	A variant of precocious sexual maturation in girls with intermediate clinical features between IPT and CPP. Breast development without pubic or axillary hair and accelerated growth velocities or advanced bone age. Pelvic ultrasound assessment shows ovaries and uteri intermediate between those of premature thelarche and precocious puberty. At LHRH test: values of FSH higher than LH, with no significant gonadotropin pulsatility. Treatment with LHRHa does not decrease breast development.	(9)
Exaggerated thelarche	Characteristics intermediate between IPT and CPP: firmer and larger breast tissue than in premature thelarche; evidence of systemic estrogen effect (bone age advancement and/or growth acceleration); sometimes ovarian enlargement. The response to the LHRH test exhibits an FSH-predominant gonadotropin response; the LH response is modest and non-significantly higher. Estradiol is increased if compared to isolated thelarche, and lower (50%) if compared to true precocious puberty. No progression or regression of their breast development and other pubertal signs after a follow-up period of at least 1 year.	(10)
	Thelarche with advanced bone age and no other features of CPP. FSH dominant response to LHRH test, with undetectable estradiol. The predominance of FSH secretion over LH may explain the self-limited nature of this condition (FSH alone is unable to sustain ovarian maturation). No data about long-term outcomes (height, gonadal status).	(11)
Unsustained central precocious puberty	Excessive stimulation of LH and FSH by LHRH test, elevated estradiol levels (62–122 pmol/L), advanced bone age (1.8–2.8 years), growth acceleration, follicular ovarian cysts at ultrasound. Outcome: transient and regressing or fluctuating.	(12)
Non-classical (atypical) premature thelarche	Non-cyclic/persistent breast development with age of onset >2 years, occasional uterine withdrawal bleeding. Predominantly FSH secretion, unsuccessful therapy with LHRHa. Occasional progression to central precocious puberty.	(13)

CPP, central precocious puberty; FSH, follicle-stimulating hormone; LH, luteinizing hormone; LHRH, LH-releasing hormone; LHRHa, LHRH analog; PT, premature thelarche.

While in CPP there is a clear activation of the HPG axis which results in the synthesis and episodic secretion of both FSH and LH and which leads to a peak LH response >5 IU/L to LHRH test (1–3), TV is characterized by an FSH-predominant response and without a peak LH response >5 IU/L to LHRH test (9–13). These features have been reported in the literature over the last decades, mainly based on small case series; however, a consensus on a unique definition was never reached. A conservative approach—without the use of LHRH analogs (LHRHa)—is usually recommended since this condition seems not to affect final height (14). Nevertheless, the clinical, laboratory, and imaging features of this condition are not univocal, and data are lacking on long-term outcomes.

This retrospective study aims to investigate the prevalence and the clinical, laboratory, and imaging features of TV in a cohort of girls with thelarche before the age of 8 years with advanced bone age and/or acceleration of growth who underwent an LHRH test. Considering the heterogeneous literature definitions of thelarche variant (TV), we relied on the laboratory diagnostic criteria only. Since normal values for stimulated FSH in prepubertal girls is 5–20 IU/L (12), we defined TV cases in which CPP was excluded (peak LH at LHRH test <5 IU/L), but peak FSH was ≥20 IU/L.

## Materials and methods

This is a retrospective cohort study on girls aged below 8 years with early thelarche (Tanner stage ≥2), with advanced bone age (bone age > chronological age + 1 year) and/or acceleration of growth (growth velocity >+2 SDS) (8) who underwent LHRH stimulation test from January 2019 to December 2022 at the Endocrinology Department of the Institute for Maternal and Child Health IRCSS “Burlo Garofolo” in Trieste, Italy.

For every selected patient, auxological (height, target height, height velocity), laboratory (basal/peak LH and FSH, time of peaks, estradiol), and imaging (bone age, uterine body-to-cervix ratio, ovarian volume) features were collected from the “G2 clinico” platform (management system specialist activities).

The height, BMI, and height velocity SDS were determined by employing Growth Calculator 3 Software. Height and BMI SDS were assessed according to the Italian reference (15), whereas height velocity SDS were assessed with the Tanner charts (16).

Stimulation tests with LHRH (0.1 mg/m^2^ Relefact LH-Releasing Hormone, Sanofi-Aventis, Frankfurt am Main, Germany) were performed according to protocols, as previously described (17). Serum LH and FSH levels were measured at baseline and at different times after stimulation (15, 30, 45, 60, 90, and 120 min) and were determined by immunochemiluminometric assay (ICMA). The kits used were FSH IRMA kit and Access hLH kit, both compatible with Beckman Coulter DxI 9000. The hormones had an intra-assay coefficient of variation below or equal to 4.05% and an inter-assay coefficient of variation below or equal to 8.2%. For the FSH hormone, the analytical sensitivity was 0.2 IU/L for the lowest level to the highest calibrator of 80 IU/L. Instead, the LH hormone lowest detectable level distinguishable from zero (Access hLH Calibrators) with 95% confidence was 0.1 IU/L. According to the results of the LHRH stimulation test, we defined CPP when peak LH was ≥5 IU/L, TV when peak LH was <5 IU/L but peak FSH was ≥20 IU/L, and PT when both peak LH was <5 IU/L and peak FSH <20 IU/L.

Serum estradiol was measured by using a competitive chemiluminescent immunoassay using coated magnetic particles. The kit was E2 IMMULITE^®^ 2000 System with a coefficient of variation of 9.7% at 389 pmol/L, 7.4% at 1020 pmol/L, and 8.5% at 2067 pmol/L and a lowest detectable level of 44 pmol/L.

Bone age was evaluated according to Greulich and Pyle by an experienced pediatric endocrinologist (18).

Transabdominal 2D gray-scale pelvic ultrasound (US) was performed by a US expert gynecologist with more than 25 years of experience using a Voluson E10 (General Electric Healthcare GE, Zipf, Austria), with a 1–5-MHz transabdominal transducer with a full bladder. The uterus was visualized along the longitudinal, transverse, and anteroposterior axes to calculate the volume and the ratio between the height of the body and the uterine cervix. Endometrial echogenicity and thickness were evaluated and reported. Longitudinal, transverse, and anteroposterior axes of the ovaries were calculated to determine the volume. The number of the follicles with diameter >5 mm was documented.

Ethical Committee approval was not requested since General Authorization to Process Personal Data for Scientific Research Purposes (Authorization no. 9/2014) declared that retrospective archive studies that use ID codes, preventing the data from being traced back directly to the data subject, do not need ethics approval (19). Informed consent was signed by parents at the first visit, in which they agreed that “clinical data may be used for clinical research purposes, epidemiology, study of pathologies and training, with the objective of improving knowledge, care and prevention”.

All data were collected in an anonymous database. All statistical analyses were conducted with JMP™ (version 16.1.0, SAS Institute Inc., Cary, NC, United States). Descriptive statistics was used to describe data. Continuous variables were expressed as median with interquartile range, minimum, and maximum. Categorical data were expressed as percentages (%). Mann–Whitney rank-sum tests and two-tailed Fisher exact tests were performed to evaluate the relations between variables. Statistical significance was considered for p-values <0.05.

## Results

**Figure 1:**
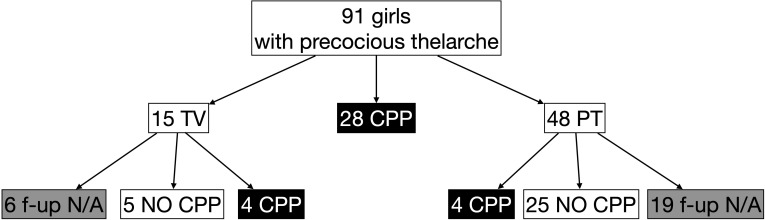
Prevalence of central precocious puberty (CPP) [peak LH ≥5 IU/L], thelarche variant (TV) [peak LH <5 IU/L but peak FSH ≥20 IU/L], and premature thelarche (PT) [both peak LH <5 IU/L and peak FSH <20 IU/L] in girls with early thelarche (<8 years) with advanced bone age (bone age > chronological age + 1 year) and/or acceleration of growth (growth velocity >+2 SDS) who performed LHRH stimulation test. f-up N/A: follow-up not available.

During the study period, a total of 104 LHRH stimulation tests were performed in 91 girls (81 girls were assessed once, 7 girls twice, and 3 girls thrice), with a median age at the time of assessment of 7.4 years (IQR 6.4;7.8; minimum 1.6, maximum 7.9]. In 54 cases (52%), bone age was advanced more than 1 year compared with chronological age; in 52 cases (50%), growth velocity was >+2 SDS. Using the laboratory parameters, as aforementioned, and considering the first stimulation test in case of multiple tests, the prevalence of TV was 16%, CPP was 31%, and PT was 53% (**Figure 1**).

Clinical, laboratory, and imaging features of the entire cohort and the three groups are presented in **Table 2**.

**Table 2:** Clinical, laboratory, and imaging features of the entire cohort and the three groups.

	Entire cohort	Thelarche variant (TV)	Premature thelarche (PT)	p TV vs PT	Central precocious puberty (CPP)	p TV vs CPP	p PT vs CPP	p
*N (%)*	104 (100%)	18 (17%)	54 (52%)		32 (31%)			
*Age (years)*	7.4 (6.4;7.8) [1.6÷7.9]	5.5 (4.7;7.1) [1.6;7.8]	7.5 (7.0;7.8) [5.7÷7.9]	**<0.01**	7.5 (7.0;7.8) [5.7÷7.9]	**<0.01**	0.81	**<0.01**
*Height (SDS)*	1.0 (0.6;1.8) [0.0÷3.2]	0.7 (0.4;1.2) [0.1÷2.8]	1.1 (0.6;1.8) [0.0÷3.2]	0.10	0.9 (0.5;1.9) [0.1÷2.4]	0.26	0.74	0.28
*Target height (SDS)*	0.7 (0.2;1.1) [0.0÷3.8]	0.7 (0.1;0.8) [0.0;3.8]	0.7 (0.2;1.1) [0.0÷3.2]	0.48	0.7 (0.1;1.1) [0.1÷3.3]	0.70	0.55	0.70
*Height – target height (SDS)*	0.2 (-0.3;1.0) [-2.5÷2.6]	0.1 (-0.7;1.0) [-2.2÷1.7]	0.1 (-0.2;1.0) [-0.6÷2.6]	0.35	0.5 (-0.3;1.0) [-2.5÷2.2]	0.36	0.91	0.60
*Height velocity (SDS)*	3.0 (1.3;4.5) [0.1÷24.7]	2.1 (1.1;3.6) [0.5÷7.7]	2.8 (1.0;4.5) [0.1÷24.7]	0.57	3.2 (1.9;5.7) [0.3÷6.4]	0.18	0.32	0.38
*BMI (SDS)*	0.8 (0.3;1.1) [0.0÷2.2]	0.6 (0.2;0.9) [0.0;3.8]	0.8 (0.3;1.2) [09.0÷2.2]	0.21	0.8 (0.3;1.2) [0.0÷2.1]	0.15	0.66	0.33
*Bone age (years)*	8.0 (7.5;9.5) [2.0÷12.0]	6.0 (5.4;7.8) [2.0÷9.0]	8.7 (7.8;9.9) [5.8÷11.0]	**<0.01**	8.8 (7.8;10.0) [5.7÷12.0]	**<0.01**	0.15	**<0.01**
*Bone age – chronological age (years)*	1.2 (0.5;2.1) [-1.5÷4.5]	0.8 (0.3;1.2) [-0.3÷2.6]	1.4 (0.6;2.1) [-1.1÷3.2]	**0.04**	1.4 (0.8;2.8) [-1.5÷4.5]	**0.02**	0.32	0.05
*Basal LH (IU/L)*	0.2 (0.2;0.4) [0.1÷2.8]	0.2 (0.2;0.2) [0.2÷1.3]	0.2 (0.2;0.2) [0.1;0.8]	0.51	0.6 (0.2;1.5) [0.2÷2.8]	**<0.01**	**<0.01**	**<0.01**
*Basal FSH (IU/L)*	2.2 (1.5;3.1) [0.2÷11.4]	2.5 (1.9;3.1) [1.6÷4.8]	1.9 (1.1;2.5) [0.2÷4.1]	**<0.01**	3.4 (2.1;5.6) [0.2÷11.4]	0.05	**<0.01**	**<0.01**
*Basal LH/FSH ratio*	0.13 (0.08;0.20) [0.04÷14.00]	0.08 (0.06;0.12) [0.04÷0.76]	0.13 (0.09;0.19) [0.04÷1.00]	**0.01**	0.15 (0.11;0.24) [0.05÷14.00]	**<0.01**	0.14	**<0.01**
*Peak LH (IU/L)*	3.7 (2.4;6.8) [0.3÷66.2]	3.1 (2.7;3.9) [2.3÷4.5]	2.7 (1.6;3.7) [0.3÷5.0]	0.07	14.6 (7.5;26.3) [5.1÷66.2]	**<0.01**	**<0.01**	**<0.01**
*Time peak LH (min)*	45 (30;45) [15÷90]	45 (30;60) [30÷90]	45 (45;60) [30÷90]	0.27	30 (30;45) [15÷90]	**0.03**	**<0.01**	**<0.01**
*Peak FSH (IU/L)*	14.5 (11.6;20.5) [1.7÷57.0]	26.6 (21.1;32.5) [20.1÷47.1]	12.8 (10.0;14.6) [1.7÷18.8]	**<0.01**	16.2 (12.8;22.1) [2.2÷57.0]	**<0.01**	**<0.01**	**<0.01**
*Time peak FSH (min)*	90 (60;90) [30÷120]	60 (60;90) [45÷120]	90 (90;120) [45;120]	**<0.01**	75 (45;90) [30÷120]	0.99	**<0.01**	**<0.01**
*Peak LH/FSH ratio*	0.23 (0.14;0.58) [0.07÷6.27]	0.11 (0.08;0.15) [0.07÷0.21]	0.18 (0.14;0.29) [0.08÷0.92]	**<0.01**	0.85 (0.54;1.50) [0.20÷6.27]	**<0.01**	**<0.01**	**<0.01**
*Estradiol (pmol/L)*	90 (72;120) [45÷360]	88 (71;165) [55÷360]	88 (65;108) [45÷297]	0.76	103 (74;153) [47÷261]	0.81	0.36	0.68
*Undetectable estradiol (%)*	52%	52%	61%	0.53	35%	0.24	**0.02**	0.07
*Uterus length (cm)*	2.8 (2.3;3.7) [0.6÷5.3]	2.7 (2.0;2.9) [1.5÷3.9]	2.8 (2.3;3.4) [0.6÷4.6]	0.31	3.5 (2.5;4.0) [1.1÷5.3]	**0.01**	**0.02**	**0.01**
*Uterine body-to-cervix ratio >1 (%)*	58%	35%	51%	0.26	82%	**<0.01**	**<0.01**	**<0.01**
*Maximum ovarian volume (cm^3^)*	1.7 (1.1;2.6) [0.2;6.1]	1.0 (0.6;1.6) [0.4÷2.7)	1.7 (1.1;2.7) [0.3÷5.7]	**<0.01**	1.9 (1.5;2.9) [0.9÷6.1]	**<0.01**	0.09	**<0.01**
*Presence of ovarian follicle >5 mm (%)*	40%	42%	29%	0.41	56%	0.41	**0.03**	0.09

Data are expressed as median (IQR) or %.Significant p values in bold.

Girls with TV (median age 5.51 years) were younger than those with both PT (7.49 years, p < 0.01) and CPP (7.52 years, p < 0.01). A less advanced bone age (0.8 years) was found in TV compared with both PT (1.4 years, p = 0.04) and CPP (1.4 years, p = 0.02), whereas no differences were found in growth velocity among groups (median 2.1 SDS in TV, 2.8 in PT, 3.2 in CPP, p = 0.38). Moreover, no differences were detected in height velocity SDS, height SDS, target height SDS, the discrepancy between height and target height in SDS, and BMI SDS.

At baseline, CPP had a significantly higher basal LH (0.6 IU/L) compared with both TV (0.2, p < 0.01) and PT (0.2, p < 0.01); PT had lower basal FSH (1.9 IU/L) compared with both TV (2.5 IU/l, p < 0.01) and CPP (3.4 IU/L, p < 0.01), whereas no differences were found in basal FSH between TV and CPP (p = 0.05). An FSH level ≤2.41 IU/L had a sensitivity of 67.7% and a specificity of 71.4% to identify PT. The basal LH/FSH ratio was significantly lower in TV (0.08) compared with both PT (0.13, p = 0.01) and CPP (0.15, p < 0.01). A basal LH/FSH ratio <0.10 had a sensitivity of 55.6% and a specificity of 73.34% to identify TV.

At LHRH test, as expected per definition, CPP presented higher levels of peak LH (14.6 IU/L) than TV (3.1 IU/L, p < 0.01) and PT (2.7 IU/L, p < 0.01), with an earlier peak (30 min in CPP, vs. 45 min in both TV and PT, p < 0.01), whereas TV showed higher levels of peak FSH (26.6 IU/L) than PT (12. 8 IU/L, p < 0.01) and also CPP (16.2 IU/L, p < 0.01), with an earlier peak (60 min in TV, vs. 75 min in CPP and 90 min in PT). The peak LH/FSH ratio was significantly lower in TV (0.11) compared with both PT (0.18, p < 0.01) and CPP (0.85, p < 0.01).

While the rate of undetectable estradiol levels was higher in PT (61%) than in CPP (35%, p = 0.02), no significant differences between estradiol levels—when detectable—were found between the three groups (median 24 pg/mL in TV and PT, 28 pg/mL in CPP).

The pelvic ultrasound showed no significant differences in uterus length between TV (2.7 cm) and PT (2.8 cm), whereas CPP had a significantly longer uterus (3.5 cm, p = 0.02 vs. PT, p = 0.01 vs. TV). Similarly, the rate of cases in which the uterine body-to-cervix ratio was >1 was similar in PT (51%) and TV (35%) but significantly higher in CPP (82%, p < 0.01). The maximum ovarian volume was smaller in TV (1.0 cm^3^) than in both PT (1.7 cm^3^, p < 0.01) and CPP (1.9 cm^3^, p < 0.01), whereas the presence of ovarian follicle >5 mm was similar in TV (42%) and CPP (56%, p = 0.41), being reduced in PT compared with CPP (29%, p = 0.03).

Considering the 63 girls not diagnosed with CPP at the first (or single) test, we retrieved data on the follow-up of 38 girls (60%) with a median age at the last follow-up of 8.2 years (IQR 7.8;8.6) and a length of follow-up of 1.1 years (IQR 0.6;2.0) (**Figure 1**). Among the nine girls with TV, four developed CPP (44%) at a median age of 6.75 years (IQR 6.15;7.95) and with a follow-up length of 1.1 years (IQR 1.0;1.6), whereas 4 girls out of 29 firstly diagnosed with PT developed CPP (14%, p = 0.04) at a median age of 7.9 years (IQR 7.8;9.2, p = 0.14) and a median length of follow-up of 0.5 years (IQR 0.4;1.3, p = 0.14) (**Table 3**).

**Table 3:** Characteristics at baseline among girls with isolated premature thelarche (PT) and thelarche variant (TV) who progressed or not into central precocious puberty (CPP) during follow-up (available in 38 out of 63 girls).

		Progression to CPP	No progression to CPP	p
*Initial diagnosis*	*TV*	4/9 (44%)	5/9 (55%	**0.04**
	*PT*	4/29 (14%)	25/29 (86%)	
*Basal FSH (IU/L)*		3.2 (2.1;3.6)	1.9 (1.1;2.3)	**<0.01**
*Basal LH/FSH ratio*		0.07 (0.06;0.10)	0.12 (0.09;0.18)	**<0.01**
*Peak LH (IU/L)*		4.1 (2.9;4.5)	2.8 (1.6;3.6)	**0.02**

Data are expressed as median (IQR) or number (%).Significant p values in bold.

Among the 30 girls who did not develop CPP at last follow-up, no differences were found in age at last visit between the 5 girls with TV [8.4 years (IQR 6.0;10.4)] and the 25 girls with PT [8.3 years (IQR 7.8;8.6), p = 0.52], whereas follow-up was significantly longer in TV [median 3.4 years (IQR 2.3;3.9)] than in PT [median 1.1 years (IQR 0.6;1.6), p < 0.01].

At first evaluation, those who progressed to CPP had a higher basal FSH [median 3.2 IU/L (IQR 2.1;3.6)], a lower LH/FSH ratio [median 0.07 (IQR 0.06;0.10)], and a higher peak LH [median 4.1 IU/L (IQR 2.9;4.5)] compared with those who did not progress to CPP [median basal FSH 1.9 IU/L (IQR 1.1;2.3), p < 0.01; median basal LH/FSH ratio 0.12 (IQR 0.09;0.18), p < 0.01; median peak LH 2.8 IU/L (IQR 1.6;3.6); p=0.02] (**Table 3**). Both a cutoff limit of basal FSH <2.41 IU/L and basal LH/FSH ratio <0.10 had a sensitivity of 76.7% and a specificity of 75.0% to predict an evolution in CPP.

## Discussion

In the present study, we described for the first time the characteristics of thelarche variant (TV) by using an objective-only parameter at LHRH test (peak FSH ≥20 IU/L and peak LH <5 IU/L) in a cohort of 91 girls with early thelarche (median age 7.4 years), with accelerated growth (median height velocity 3.0 SDS) and advanced bone age (median +1.2 years compared with chronological age) (8) who performed a total of 104 LHRH tests: 17% fitted the definition of TV, 31% of CPP, and 52% of PT.

We found that girls with TV were younger (median 5.5 years) and with less advanced bone age (median +0.8 years) compared with both PT and CPP, probably due to the younger age itself. No differences were found in growth velocity (median 2.1 SDS) and the other auxological parameters. Pelvic ultrasound and estradiol levels were found not to be relevant in discriminating TV from PT and CPP, although TV showed a similar prevalence of presence of ovarian follicle >5 mm (42%) with CPP (56%, p = 0.41) but significantly higher than PT (29%, <p.0.01), whereas maximum ovarian volume was smaller in TV (1.0 cm^3^) than in both PT and CPP (1.7 cm^3^ and 1.9 cm^3^, respectively, p < 0.01).

Interestingly, apart from peak FSH (included in the definition itself) that was higher in TV (26.6 IU/L) not just compared with PT (12.8 IU/L) but also CPP (16.2 IU/L), even basal FSH was higher in TV (2.5 IU/L) and CPP (3.4 IU/L) compared with PT (1.9 IU/L, both p < 0.01). Since normal values for FSH in girls with Tanner stage 1 are 0.50–2.41 IU/L (20), a cutoff limit of FSH <2.41 IU/L could help in differentiating PT from other forms of early thelarche, although sensitivity and specificity are low (67.7% and 71.4%, respectively). Both basal and peak LH are similar in TV (0.2 and 3.1 IU/L, respectively) and PT (0.2 and 2.7 IU/L, respectively), but significantly lower than CPP (0.6 and 14.6 IU/L, respectively, p < 0.01).

Moreover, both basal and peak LH/FSH ratios (0.08 and 0.11, respectively) were significantly lower in TV, compared with both PT (0.13 and 0.18, respectively) and CPP (0.15 and 0.85, respectively). A cutoff limit of basal LH/FSH <0.10 in diagnosing TV also has low sensitivity and specificity (55.6% and 73.34%, respectively).

Remarkably, in those who performed a follow-up visit (60% of the 63 girls without CPP at baseline, with median age at the last visit of 8.2 years and length of follow-up of 1.1 years), only 14% of PT developed CPP [as previously reported in the literature (6)] compared with 44% of TV (p=0.04). Girls with TV had a subsequent diagnosis of CPP at an earlier age (6.75 years) compared with PT (7.9 years), although the difference did not reach statistical significance, probably due to the reduced number of individuals considered in this analysis.

At first evaluation, those who progressed to CPP had a higher basal FSH (median 3.2 IU/L), a lower LH/FSH ratio (median 0.07), and a higher peak LH (median 4.1 IU/L) compared with those who did not progress to CPP (median basal FSH 1.9 IU/L, p < 0.01; basal LH/FSH ratio 0.12, p < 0.01; peak LH 2.8 IU/L, p = 0.02). Both a cutoff limit of basal FSH <2.41 IU/L and basal LH/FSH ratio <0.10 had a sensitivity of 76.7% and a specificity of 75.0% to predict an evolution in CPP.

All these findings are in line with previous reports of an FSH-predominant gonadotropin response in TV (10, 11). It is known that in girls FSH starts to increase earlier than LH due to its role in stimulating the growth and maturation of ovarian follicles (3). As the ovarian follicles mature and start producing estrogens, thelarche develops. However, estrogens exert negative feedback on the hypothalamus and pituitary gland, which is more pronounced on LH than on FSH secretion, leading to a greater delay in the rise of LH compared with FSH (21). Therefore, the rise in FSH secretion can be seen as a “prepuberty”, an “introduction” to proper puberty. Nonetheless, FSH alone is unable to start puberty and sustain ovarian maturation. Only when the ovarian follicles have matured and estrogen levels have risen sufficiently, the negative feedback on LH is lifted, resulting in the start of a sustained puberty (22).

Although this study was not prospective in its nature, our data support the chance for TV to evolve in CPP [as already reported (7, 13)], with a higher rate (44%) compared with PT (14%) (6).

Therefore, TV might be considered a “precocious prepuberty”. On the one hand, the predominance of FSH secretion (both basal and after LHRH stimulation) may initially limit the LH secretion and the progression into proper puberty. On the other hand, it may eventually trigger a full puberty, as underlined by higher basal FSH, a lower basal LH/FSH ratio, and a higher peak LH (although <5 IU/L) in girls who developed CPP during the follow-up.

Some limitations of this study should be considered. Firstly, our data are referred to a single center, so the generalizability is limited; furthermore, the retrospective nature of the analysis does not allow the possibility of monitoring the follow-up in all girls, and to collect final height and age at menarche of, establishing with certainty the long-term outcomes of this condition. A prospective study on this aim is missing so far and would be necessary. Moreover, genetic studies in TV would help in understanding whether this “precocious prepuberty” could be linked to mutations in the same genes known to cause CPP (e.g., *GPR54*, *KISS1, MKRN3, DLK1*) (3, 23).

To our knowledge, however, this is the first study that classifies thelarche variant with laboratory objective criteria and that provides details on clinical, laboratory, and imaging features of this intermediate condition. A further study with a reassessment at the end of the growth is planned to evaluate the outcome of TV. Moreover, a prospective study on the prediction of basal FSH and LH/FSH ratio cutoff to distinguish precocious thelarche who will progress into CPP is needed.

## Data availability statement

The raw data supporting the conclusions of this article will be made available by the authors, without undue reservation.

## Ethics statement

Ethical Committee approval was not requested since General Authorization to Process Personal Data for Scientific Research Purposes (Authorization no. 9/2014) declared that retrospective archive studies that use ID codes, preventing the data from being traced back directly to the data subject, do not need ethics approval. Informed consent was signed by parents at the first visit, in which they agreed that “clinical data may be used for clinical research purposes, epidemiology, study of pathologies and training, with the objective of improving knowledge, care and prevention”.

## Author contributions

FBur: Conceptualization, Methodology, Data curation, Investigation, Writing – original draft. BL: Conceptualization, Data curation, Investigation, Methodology, Writing – original draft. GTa: Conceptualization, Methodology, Writing – original draft, Supervision. AF: Investigation, Writing – review & editing. FBuo: Investigation, Writing – review & editing. MP: Data curation, Writing – original draft. VV: Investigation, Writing – review & editing. GV: Investigation, Writing – review & editing. EF: Writing – review & editing, Supervision. EB: Supervision, Writing – review & editing. GTo: Conceptualization, Formal Analysis, Methodology, Supervision, Writing – review & editing.
